# The complete mitogenome and phylogenetic analysis of *Acrossocheilus jishouensis* (Osteichthyes: Cyprinidae)

**DOI:** 10.1080/23802359.2017.1372726

**Published:** 2017-09-08

**Authors:** Xiao-Xiang Liu, Ri-Lin Liu, Wen-Jing Ye, Xiao-Xiang Xu, Xue-Lin Song, Le-Yang Yuan

**Affiliations:** a Institute of Hydrobiology, Chinese Academy of Sciences, Wuhan, Hubei, China;; b Faculty of Basic Medicine, Hangzhou Medical College, Hangzhou, Zhejiang, China;; c Management Bureau of Wangdongyang Alpine Wetland Nature Reserve, Jingning Lishui, China;; d University of Chinese Academy of Sciences, Beijing, China;; e Zhejiang Museum of Natural History, Hangzhou, Zhejiang, China;; f Biodiversity Research Center of Zhejiang Province, Hangzhou, Zhejiang, China

**Keywords:** Barred species, mitogenome, *Acrossocheilus jishouensis*, next generation sequencing

## Abstract

*Acrossocheilus jishouensis* is an endemic south China stream-dwelling cyprinid species. Its complete mitochondrial genome is 16,587 bp in length, consisting of 13 protein-coding genes, 22 tRNA genes (ranging from 67 bp in *tRNA^Cys^* to 76 bp in *tRNA^Leu^* and *tRNA^Lys^*), two rRNA genes (956 bp in 12S rRNA and 1673 bp in 16S rRNA), and one control region (942 bp). Its overall base composition is A: 31.2%, C: 27.6%, G: 16.2%, and T: 25.1%. The complete mitogenome of the Chinese barred species of Cpynidae could provide a basic data for further phylogenetics analysis.

*Acrossocheilus jishouensis* is a barred cyprinid species from the Yuan-Jiang of Hunan Province in China, a tributary flowing to Dongting Lake connected to the middle Yangtze River (Zhao et al. 1997). This species differs from the congenus with a combination of characters: six vertical bars on the flanks 1–2 scales wide and not beyond the lateral line in the adults, a longitudinal stripe along the lateral line, two thick lateral lobes of the lower lip, last unbranched dorsal-fin ray slim and unserrated along its posterior margin, membranes between the dorsal-fin rays oblong with black blotches. However, ontogenetic changes and/or sexual dimorphism in body colouration and mouthpart structures usually confuse researchers when delineating these barred species. Thus, combination of mitochondrial DNA data and morphological traits will help for the species identification of taxonomic validity and phylogenetic position of the species.

The sample caught from the Jinping county (Qingshui Jiang, flowing to Yuan Jiang basin) of Guizhou Province in China was deposited in the collection of the Zhejiang Museum of Natural History under the accession number ZMNH 2015030001. The complete mitogenome of *A. jishouensis* has been obtained from high-throughput sequencing on whole genomic DNA with HiSeq 2000 platform (Illumina, San Diego, CA). We used next generation sequencing to perform low-coverage whole genome sequencing according to previous protocol (Shen et al. 2016). The complete mitogenome of *A. jishouensis* is 16,587 bp in length (GenBank KY131974), includes 13 protein-coding genes, 22 tRNA genes (ranging from 67 bp in *tRNA^Cys^* to 76 bp in *tRNA^Leu^* and *tRNA^Lys^*), two rRNA genes (956 bp in 12S rRNA and 1673 bp in 16S rRNA), and one D-loop control region (942 bp). Its overall base composition is A: 31.2%, C: 27.6%, G: 16.2%, and T: 25.1% which shows AT bias, with the AT content of 57.4%. The complete mitogenome of *A. jishouensis* shows 95% identities to *A. parallens* (GenBank KP274878) after BLAST search against NCBI nr/nt database.

To validate the phylogenetic position of *A. jishouensis*, we used MEGA6 software (Tamura et al. 2013) to construct a maximum likelihood tree (with 500 bootstrap replicates and Kimura 2-parameter model) containing complete mitogenomes of 11 species derived from *Acrossocheilus* genus. *Onychostoma gerlachi* (Cheng et al. 2016) derived from *Onychostoma* genus was used as outgroup for tree rooting. The resultant phylogeny shows that the *A. jishouensis* is closely related to *A. parallens* with high bootstrap value supported (Figure 1). The resultant phylogeny showed that the sister pair *A. parallens* and *A. hemispinus* were sister to *A*. *jishouensis*, with the basal position of the group occupied by *A*. *jishouensis*. The Kimura-2-parameter distance was 0.055 between *A. jishouensis* and *A. parallens,* and was 0.057 between *A*. *jishouensis* and *A. hemispinus*, much higher than that between the sister pair *A. parallens* and *A. hemispinus* (0.022) in the same group. The complete mitogenome of *A. jishouensis* which is deduced in this study provides a basic data for further phylogenetic and conversational analysis of the Chinese barred species in Cyprinidae.

**Figure 1.**
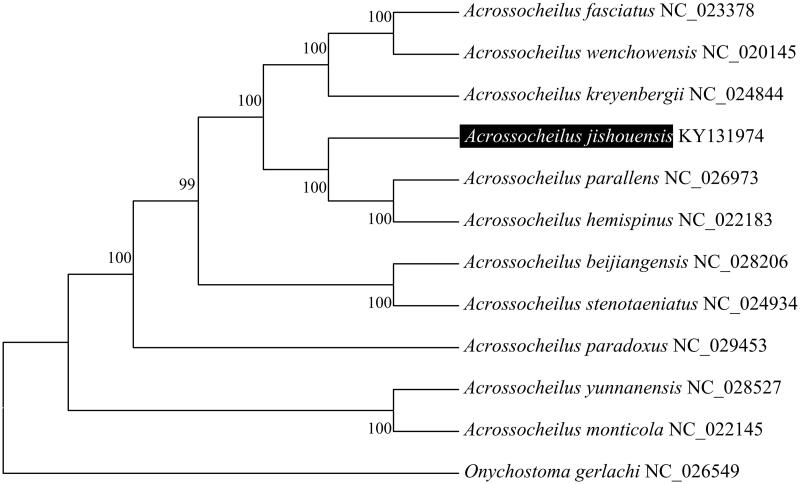
Molecular phylogeny of *Acrossocheilus jishouensis* and related species based on complete mitogenome. The complete mitogenomes is downloaded from GenBank and the phylogenic tree is constructed by Maximum likelihood method with 500 bootstrap replicates. The gene's accession number for tree construction is listed behind the species name.
